# Left ventricular apical aneurysm resection and mitral valve replacement for apical hypertrophic cardiomyopathy

**DOI:** 10.1002/ccr3.7974

**Published:** 2023-09-28

**Authors:** Hironobu Nishiori, Hiroyuki Watanabe, Yuichi Hirano, Masayoshi Otsu

**Affiliations:** ^1^ Division of Cardiovascular Surgery Narita Red Cross Hospital Chiba Japan

**Keywords:** apical hypertrophic cardiomyopathy, atrial functional mitral regurgitation, left ventricular apical aneurysm

## Abstract

**Key clinical message:**

Left ventricular apical hypertrophic cardiomyopathy with an apical aneurysm carries a risk of thrombosis and can also lead to atrial fibrillation and functional mitral regurgitation.

**Abstract:**

A 78‐year‐old woman underwent left ventricular apical aneurysm (LVAA) resection and mitral valve replacement for severe atrial functional mitral regurgitation. ApHCM can cause atrial fibrillation and atrial functional mitral valve regurgitation. LVAA resection in addition to mitral valve replacement was reasonable to prevent fatal complications associated with LVAA.

## Graphical abstract

Left ventricular apical hypertrophic cardiomyopathy with an apical aneurysm carries a risk of thrombosis and can also lead to atrial fibrillation and atrial functional mitral regurgitation.

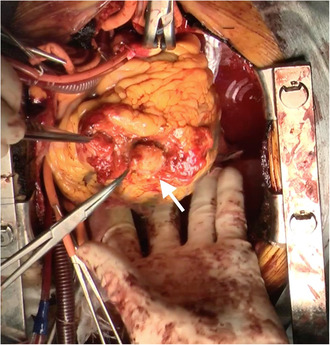

## 1 CASE

**FIGURE 1:**
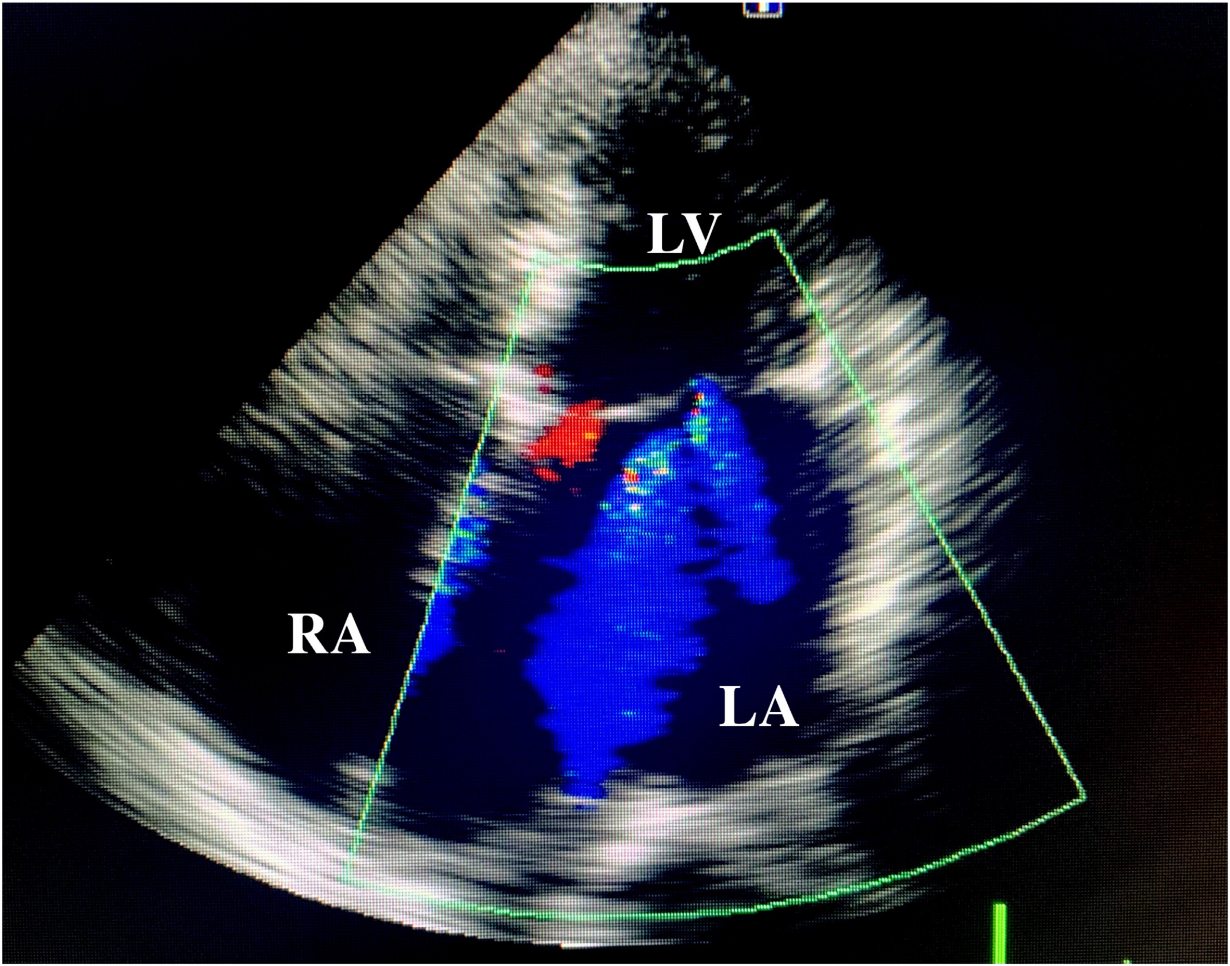
Transthoracic echocardiography imaging showing severe mitral valve regurgitation and severely dilated left atrium. LA, left atrium; LV, left ventricular; RA, right atrium.

**FIGURE 2:**
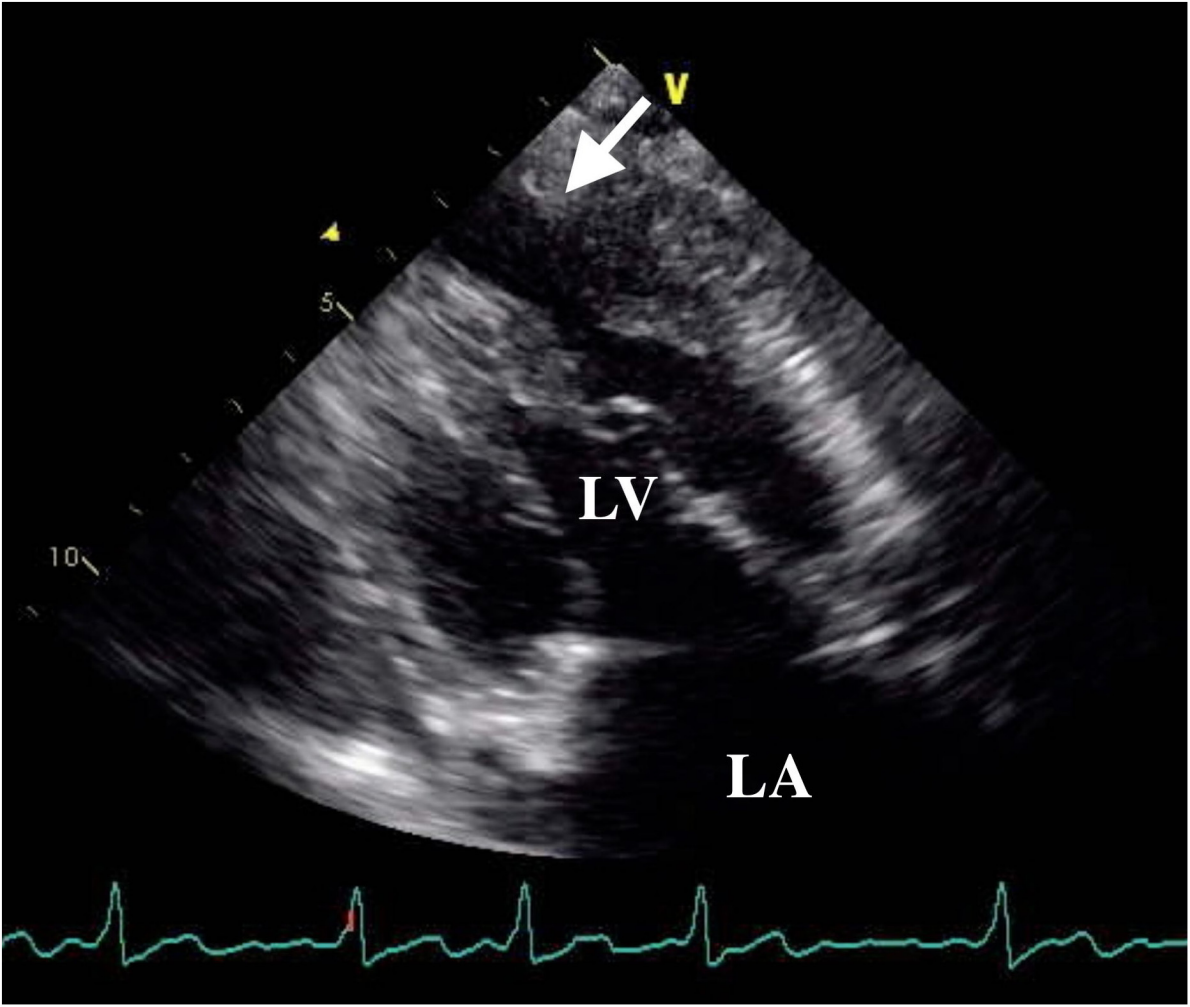
Transthoracic echocardiography imaging showing apical hypertrophic cardiomyopathy with left ventricular apical aneurysm (arrow). LA, left atrium; LV, left ventricular.

**FIGURE 3:**
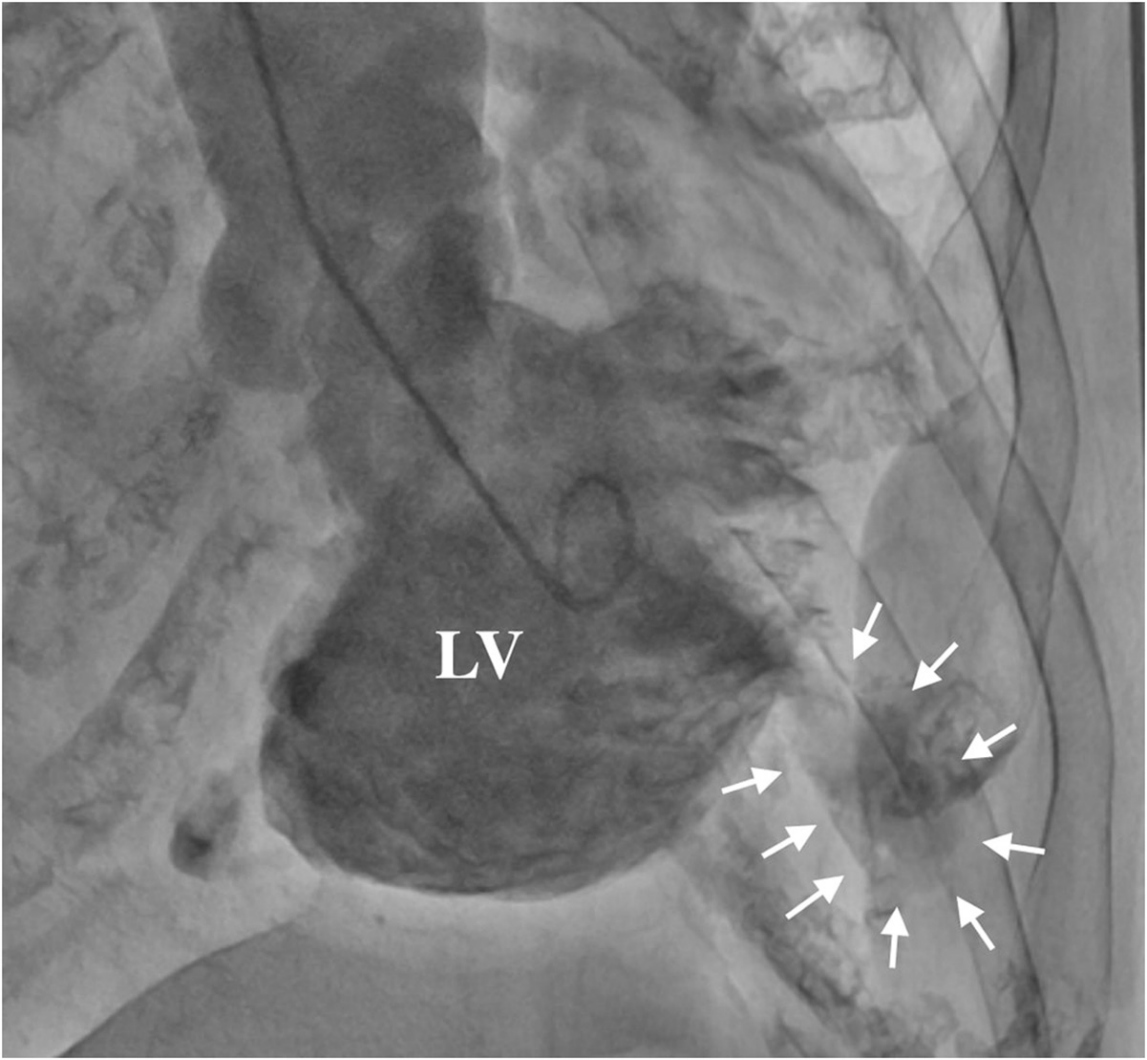
Left ventricular angiogram showing the left ventricular apical aneurysm (arrows).

**FIGURE 4:**
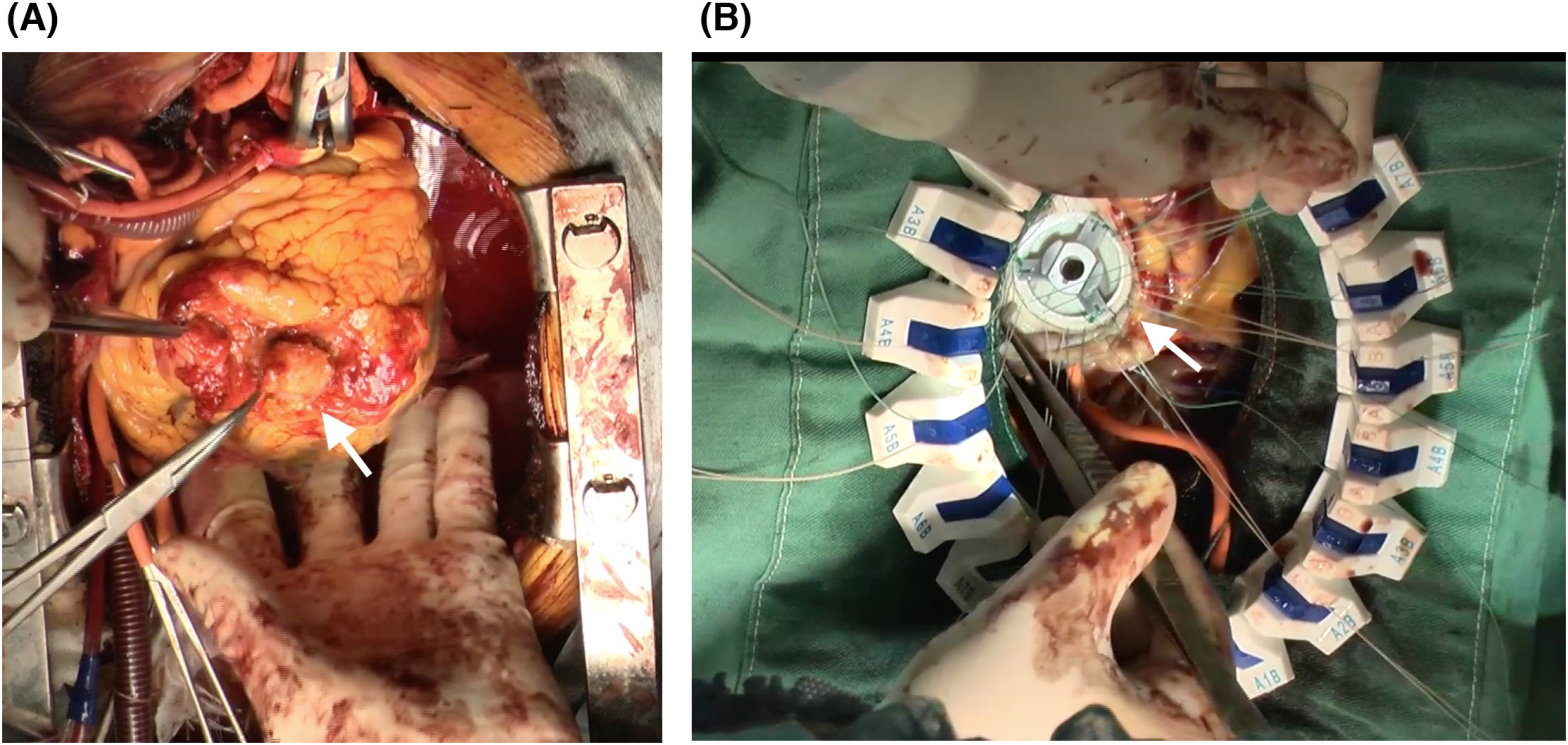
(A) Intraoperative findings indicating left ventricular pseudoaneurysm derived from left ventricular (arrow). (B) Intraoperative image of mitral valve replacement (arrow).

**FIGURE 5:**
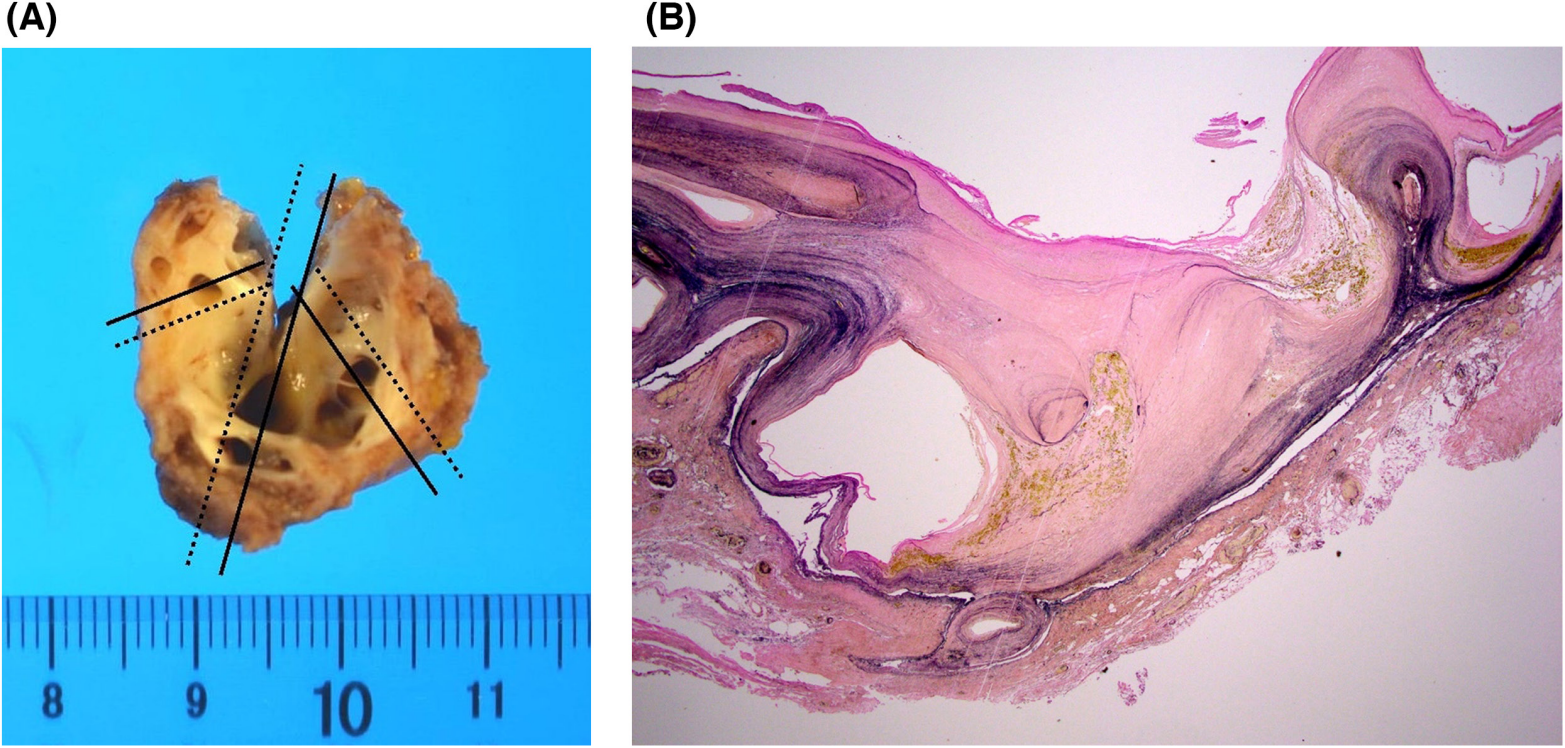
(A) The resected left ventricular apical aneurysm. (B) Elastica‐van Gieson staining of the wall of left ventricular apical aneurysm showing thick fibrosis and stretched myocardium.

A 78‐year‐old woman with histories of apical hypertrophic cardiomyopathy (ApHCM), chronic atrial fibrillation (AF), and cerebral infarction were referred to our hospital for chronic heart failure. The transthoracic echocardiography revealed severely dilated right and left atrium and severe mitral valve regurgitation (MR) with preserved ejection fraction (Figure 1). The transthoracic echocardiography and left ventriculography showed the left ventricular apical hypertrophy with a left ventricular apical aneurysm (LVAA) (Figures 2 and 3).^1^ The patient was diagnosed as severe atrial functional MR. She underwent mitral valve replacement, left appendage resection, and LVAA resection (Figure 4). Histopathological examination of the LVAA revealed the thick fibrosis and stretched myocardial cells (Figure 5A,B).

We reported a rare case of ApHCM and LVAA leading to AF and atrial functional severe MR. ApHCM can develop AF due to diastolic dysfunction, and prolonged AF may lead to atrial functional MR. Since LVAA associated with ApHCM is known to cause fatal complications including arrhythmia and thrombosis,^2^ in this case, LVAA resection was performed in addition to mitral valve replacement. Even if there were no symptoms due to the LVAA at the time of surgery, the concomitant resection will prevent these future complications especially patients with history of cerebral infarction. Herein, we report a rare case of simultaneous mitral valve replacement and LVAA resection.

## AUTHOR CONTRIBUTIONS

**Hironobu Nishiori:** Conceptualization; data curation; formal analysis; resources; software; writing – original draft; writing – review and editing. **Hiroyuki Watanabe:** Conceptualization; resources; software; supervision. **Masayoshi Otsu:** Conceptualization; supervision. **Yuichi Hirano:** Conceptualization; resources.

## FUNDING INFORMATION

None.

## CONFLICT OF INTEREST STATEMENT

The authors have no pertinent conflicts of interest to report for this manuscript.

## ETHICS STATEMENT

None.

## CONSENT

Written informed consent was obtained from the patient to publish this report in accordance with the journal's patient consent policy.

## Data Availability

Data sharing not applicable to this article as no datasets were generated or analysed during the current study.
